# USP35 activated by miR let-7a inhibits cell proliferation and NF-κB activation through stabilization of ABIN-2

**DOI:** 10.18632/oncotarget.4451

**Published:** 2015-06-26

**Authors:** Chunyan Liu, Lina Wang, Weiwen Chen, Shihu Zhao, Chunli Yin, Yani Lin, Anli Jiang, Pengju Zhang

**Affiliations:** ^1^ Department of Biochemistry and Molecular Biology, Shandong University School of Medicine, Jinan, Shandong, 250012, P.R. China

**Keywords:** ABIN-2, deubiquitylase, miR let-7a, NF-κB, USP35

## Abstract

Ubiquitin specific protease 35 (USP35) is a member of deubiquitylases (DUBs). It remains largely unknown about the biological role and the regulation mechanism of USP35. Here, we first identified miR let-7a as a positive regulator of USP35 expression and showed that USP35 expression positively correlates with miR let-7a expression in different cancer cell lines and tissues. Then, we showed that USP35 expression was decreased dramatically in the tumor tissues compared with the adjacent non-cancerous tissues. USP35 overexpression inhibited cell proliferation *in vitro* and inhibited xenograft tumor growth *in vivo*. Furthermore, we revealed that USP35 acts as a functional DUB and stabilizes TNFAIP3 interacting protein 2 (ABIN-2) by promoting its deubiquitination. Functionally, both ABIN-2 and USP35 could inhibit TNFα-induced NF-κB activation and overexpression of ABIN-2 alleviated USP35-loss induced activation of NF-κB. Collectively, our data indicated that miR let-7a-regulated USP35 can inhibit NF-κB activation by deubiquitination and stabilization of ABIN-2 protein and eventually inhibit cell proliferation. Overall, our study provides a novel rationale of targeting miR let-7a-USP35-ABIN-2 pathway for the therapy of cancer patients.

## INTRODUCTION

MicroRNA (MiR) let-7a has been widely studied and functions as a tumor suppressor [1]. Its expression is down-regulated in several cancers including lung cancer, breast cancer and prostate cancer among others [2–4]. The decreased let-7a expression is linked to increased tumorigenicity and poor prognosis. Previous work has shown miR let-7a inhibits cell proliferation by down-regulating oncogenes *RAS/c-MYC* [5] and *HMGA-2* [6] at the translational level in lung cancer cells. In prostate cancer cells, miR let-7a targets E2F2 and CCND2 to inhibit cell proliferation [4]. We previously demonstrated that miR let-7a directly targets the 3′ UTR of the *IGF1R* mRNA and inhibits its expression in prostate cancer cells [7]. Although accumulating evidences indicate that miR let-7a exerts its anti-tumor activity by regulating oncogenes or tumor suppressor genes [8], the precise mechanisms of miR let-7a in the pathogenesis of human cancers still remain unclear and the full set of its regulatory substrates need to be further elucidated.

Ubiquitination is an important post-translational modification that regulates diverse cellular events, including DNA damage response, cell cycle regulation and intracellular trafficking [9, 10]. Similar to other posttranslational modifications, ubiquitination is a reversible process [11]. There is a family of enzymes, termed deubiquitinases (DUBs) which can catalyze the removal of ubiquitin from the ubiquitinated substrates [12]. According to their enzyme features, DUBs can be divided into five classes: USPs (ubiquitin-specific proteases), UCHs (ubiquitin C-terminal hydrolases), OTUs (ovarian proteases), MJDs (Machado–Joseph disease proteases) and JAMMs (the Jab1/MPN/Mov34 metalloenzymes) [13]. The USP family represents the most abundant group within DUBs with over 60 members [14]. A growing number of studies have shown that these DUBs have a close relation with cancer development and progression due to their oncogenic and tumor suppressive functions. USP7 is overexpressed in prostate cancer where it drives the deubiquitination and nuclear exclusion of PTEN, which has been correlated with more aggressive disease [15]. USP17 was up-regulated in squamous non-small cell lung cancer (NSCLC) patients which has been correlated with the recurrence of disease at distant sites by regulating Ras pathway [16]. High expression levels of USP28 have also been reported in colon and breast carcinoma. The deubiquitinating activity of USP28 is known to stabilize the levels of the oncogenic transcription factor Myc [17]. Conversely, USP10 and USP13 are thought to function as a tumor suppressor through deubiquitination and stabilization of p53 and PTEN respectively [18, 19]. Although much progress has been made in the characterization of this DUBs family recently, the cellular roles and regulation of a large proportion of DUBs are still poorly understood.

In the present study, we first identified USP35, a member of the USP superfamily, can be up-regulated by the miR let-7a. We then determined the roles of USP35 and found that overexpression of USP35 could inhibit cancer cell proliferation in *vitro* and tumorigenesis in *vivo*. In addition, overexpression of USP35 inhibited TNFα-induced NF-κB activation by deubiquitination and stabilization of TNFAIP3 interacting protein 2 (ABIN-2). These findings indicate that USP35 up-regulated by miR let-7a potentially functions as a tumor suppressor and inhibits tumor growth. Our findings not only provide a novel molecular insight into miR let-7a-mediated tumor suppression, but also implicate USP35 as a promising molecular target for cancer therapy for the first time.

## RESULTS

### MiR let-7a up-regulates the expression of USP35

**Figure 1:**
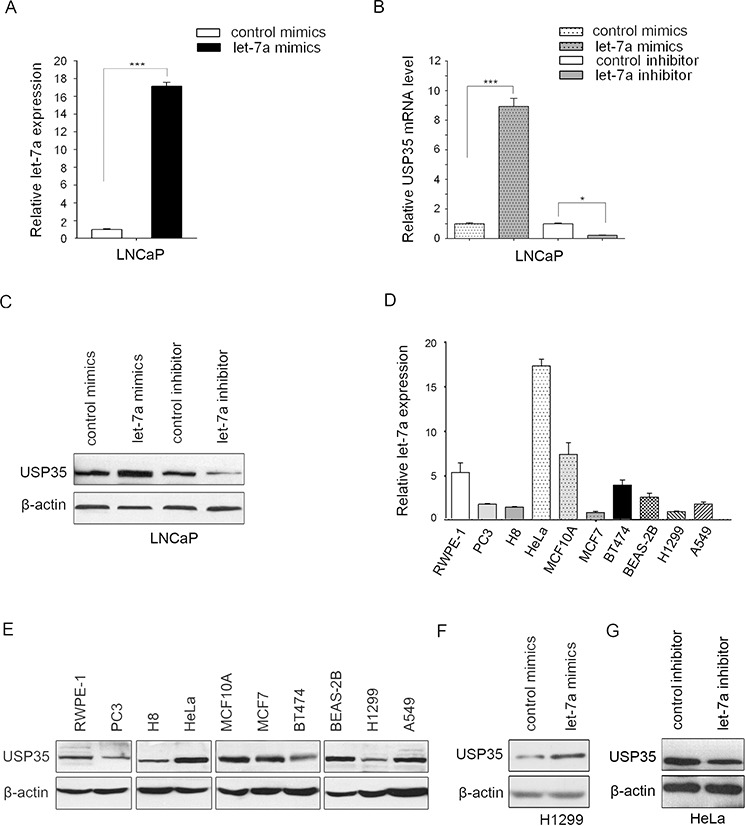
MiR let-7a up-regulates the expression of USP35 **A.** The expression of miR let-7a was examined by qRT-PCR in LNCaP cells transfected with let-7a mimics or control mimics. The results confirmed the transfection efficiency. **B.** The mRNA of USP35 was detected by qRT-PCR in LNCaP cells transfected with let-7a mimics or let-7a inhibitor. **C.** The protein of USP35 was detected by Western blot in LNCaP cells transfected with let-7a mimics or let-7a inhibitor. **D.** The expression of let-7a was measured by qRT-PCR in different cells as indicated. **E.** The protein of USP35 was measured by Western blot in the cells described in D. **F.** The protein of USP35 was detected by Western blot in H1299 cells transfected with let-7a mimics or control mimics. **G.** The protein of USP35 was detected by Western blot in HeLa cells transfected with let-7a inhibitor or control inhibitor. All results are from three independent experiments. *P < 0.05, ***P < 0.001 based on Student *t-test*.

MiR let-7a has been reported to act as a tumor suppressor in some cancer types, such as lung and prostate cancer [7, 20]. MiR let-7a exerts its antitumorigenesis roles mainly through regulating the expression of some oncogenes such as RAS and CCND2 [1]. However, a detailed understanding of the full set of targets regulated by miR let-7a still needs to be further addressed. To identify the novel targets that can be regulated by miR let-7a, we performed cDNA microarray analysis in the human prostate cancer cell line LNCaP after transfection with let-7a mimics or let-7a inhibitor. Several differentially expressed genes were identified, of which *USP35* was elevated significantly in LNCaP cells with *let-7a* mimics while decreased with let-7a inhibitor. To confirm the results obtained from analyzing the mRNA profiling, we transfected let-7a mimics or inhibitor into LNCaP cells and used qRT-PCR and Western blot to detect the expression of USP35. Consistent with the results of microarray analysis, the results showed that let-7a mimics increased, whereas let-7a inhibitor decreased both mRNA and protein level of USP35 (Figure 1A–1C).

We next examined let-7a and USP35 expression in a panel of 10 human cancer cell lines and their corresponding non-malignant cell lines. The results showed that the expression of USP35 was much lower in PC3 prostate cancer cells than that in non-malignant prostate epithelial cells RWPE-1. The expression of USP35 also decreased in MCF7 and BT474 breast cancer cells as well as in H1299 and A549 lung cancer cells compared with that in non-malignant breast epithelial cells MCF10A and that in lung epithelial cells BEAS-2B. Although the expression of USP35 showed an increase in HeLa cervical cancer cells compared with that in non-malignant cervical epithelial cells H8, its expression decreased in the most cancer cell lines. Meanwhile miR let-7a has similar expression pattern with USP35 (Figure 1D and 1E). To further validate our observation, USP35 protein level was measured after overexpression of let-7a mimics in H1299 cells (expressing lower let-7a) or let-7a inhibitor in HeLa cells (expressing higher let-7a). The results showed that miR let-7a mimics increased USP35 expression and its inhibitor decreased USP35 expression (Figure 1F and 1G). However, neither ectopic expression of USP35 in H1299 nor silenced expression of USP35 in LNCaP cells had a significant effect on the expression of miR let-7a (Supplementary Figure S1). Together, our data suggests that USP35 is a novel target of miR let-7a.

### USP35 expression is down-regulated in human cancer tissues and positively associated with miR let-7a expression

**Figure 2:**
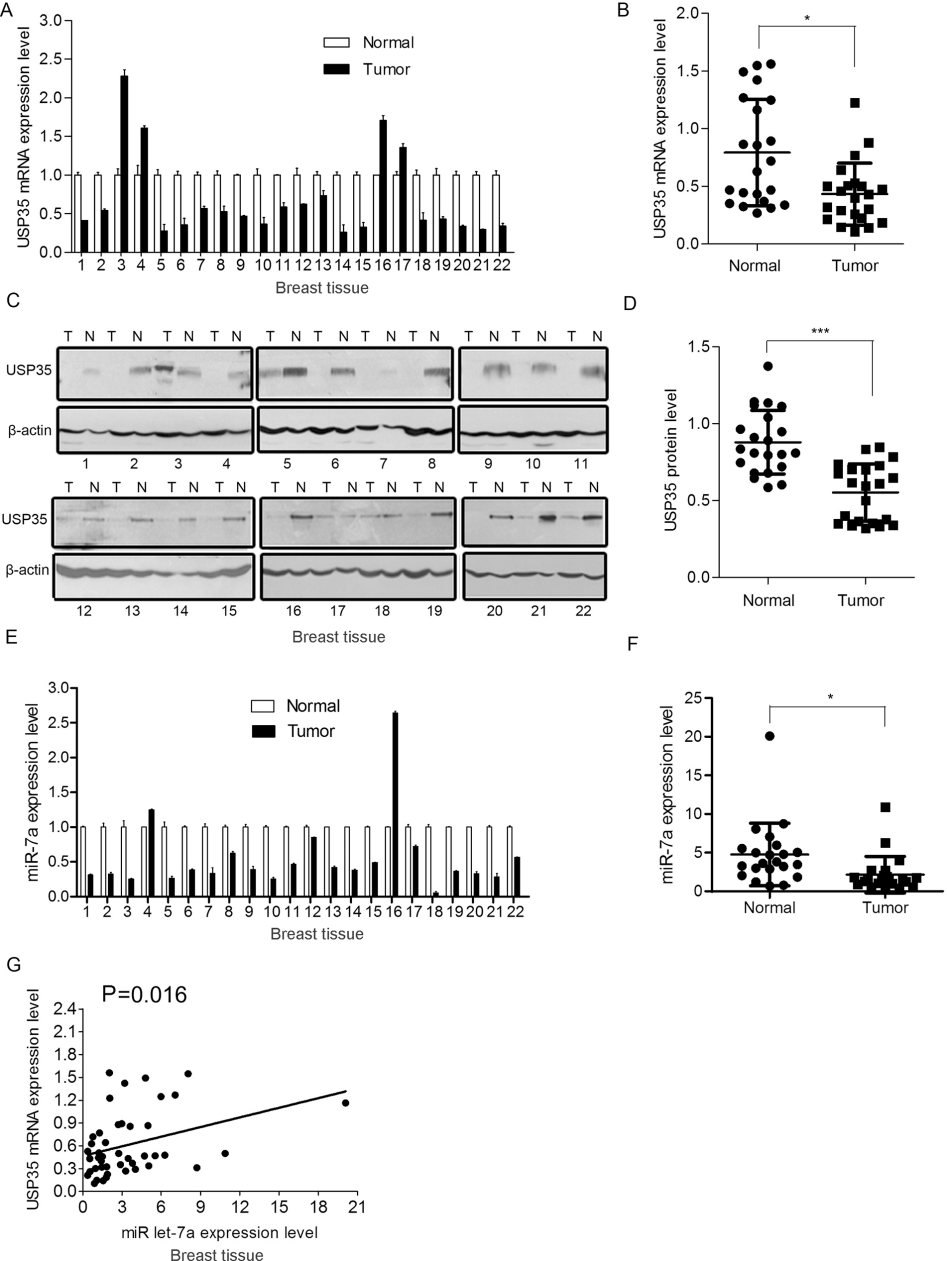
USP35 expression is decreased in cancer tissues and positively associated with miR let-7a expression **A.** The mRNA of USP35 was detected by qRT-PCR in breast cancer tissues and adjacent non-cancerous tissues. **B.** USP35 mRNA expression levels in breast cancer tissues and adjacent non-cancerous tissues (*n* = 22) were shown as scatter diagram. Data, mean ± SD. **C.** The protein of USP35 was detected by Western blot in breast cancer tissues and adjacent non-cancerous tissues. N and T: normal and tumor tissues. **D.** Relative USP35 protein expression levels in breast cancer tissues and non-cancerous tissues (*n* = 22) were shown as scatter diagram. Data, mean ± SD. **E.** The expression of miR let-7a was examined by qRT-PCR in breast cancer tissues and adjacent non-cancerous tissues. **F.** MiR let-7a expression levels in breast cancer tissues and adjacent non-cancerous tissues (*n* = 22) were shown as scatter diagram. Data, mean ± SD. **G.** Regression analysis of the correlations between the expression of USP35 and miR let-7a expression. *P < 0.05, ***P < 0.001 based on Student *t-test*.

To explore any clinical significance of the observation, we analyzed the expression of USP35 and miR let-7a in human cancer tissues and the corresponding non-cancerous normal tissues as indicated. We first examined the expression of USP35 in clinical samples using qRT-PCR and Western blot. 18/22 (81.8%) of the clinical breast cancer specimens and 9/11(81.8%) of the lung cancer specimens had lower USP35 mRNA levels than adjacent normal tissues (Figure 2A; Supplementary Figure S2A). The mean value of USP35 levels in the cancer tissues was significantly lower than that in the normal tissues (*P* < 0.05, Figure 2B; Supplementary Figure S2B). Consistent with the qRT-PCR results, the protein level of USP35 was also significantly decreased in the cancer tissues compared with the adjacent normal tissues (Figure 2C and 2D; Supplementary Figure S2C and S2D). These data demonstrated that USP35 expression is down-regulated in human cancer tissues.

Then we determined miR let-7a expression in the same set samples using qRT-PCR and examined the correlation between miR let-7a and USP35 expression in tumors. As expected, 20/22 (90.9%) of the clinical breast cancer specimens and 8/11(72.7%) of the lung cancer specimens showed decreased expression of miR let-7a (Figure 2E; Supplementary Figure S2E). The mean value of miR let-7a levels in the cancer tissues was significantly lower than that in the normal tissues (*P* < 0.05, Figure 2F; Supplementary Figure S2F). 20/22 samples showed the similar trend of miR let-7a and USP35 expression in breast tumor tissues compared with the non-cancerous tissue. Statistical analysis showed that USP35 expression was positively correlated with miR let-7a level in breast cancer tissues (*P* < 0.05) (Figure 2G).

### USP35 inhibits cancer cell proliferation

**Figure 3:**
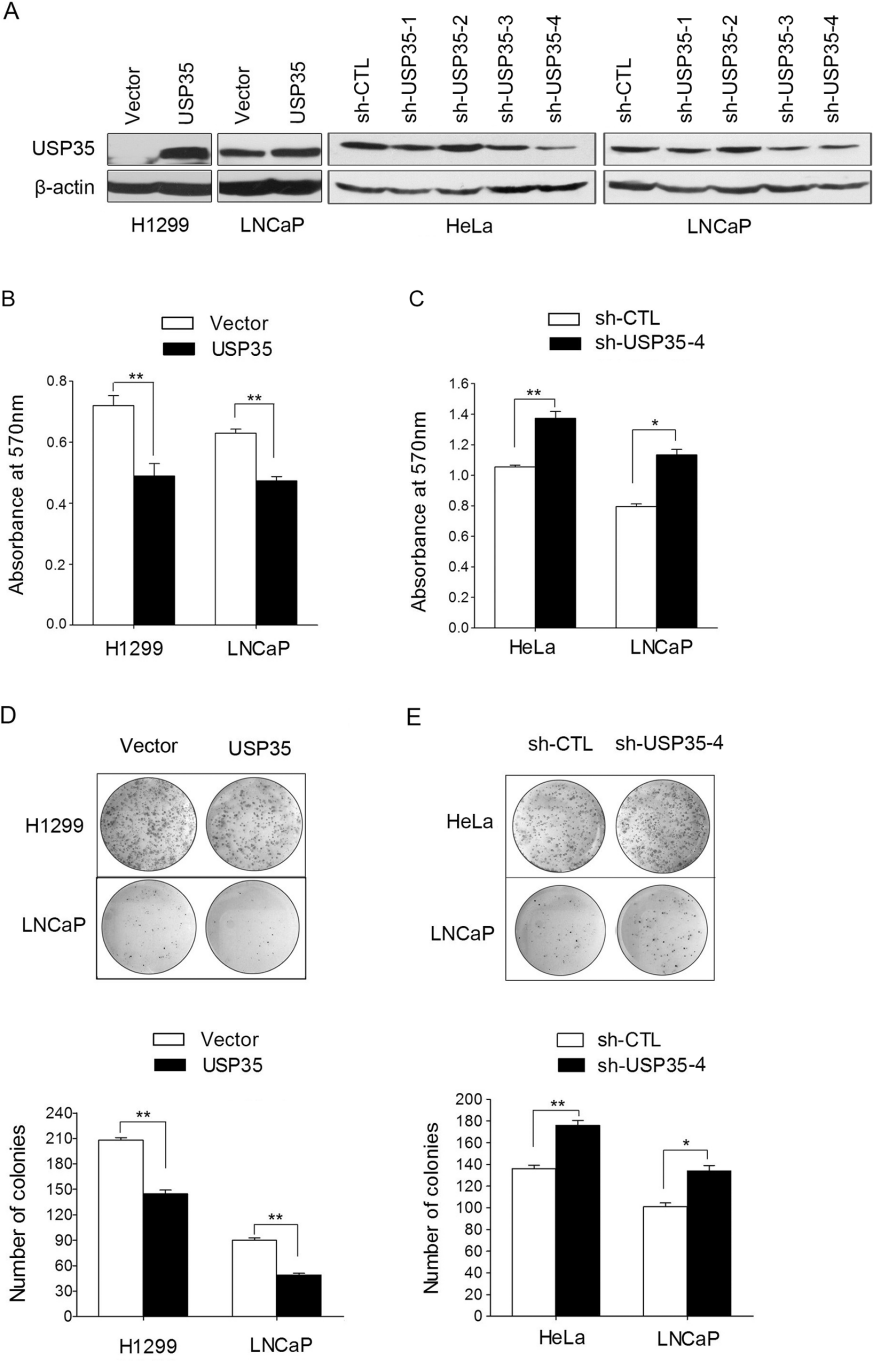
USP35 inhibits cancer cell proliferation **A.** The protein of USP35 was verified by Western blot in H1299 and LNCaP cells with ectopic USP35 expression and in HeLa and LNCaP cells with silenced USP35 expression. **B. and C.** MTT analysis was performed in the abovementioned cells. **D. and E.** Colony formation assay was performed to assess the clonogenic potentials of the abovementioned cells. The graph shows quantitative analysis (bottom). Data, mean ± SD. *P < 0.05, **P < 0.01 based on Student *t-test*.

USP35 is a member of DUB family and locates on chromosomal 11q14.1, but up to now, there is little known about the biological roles of USP35. To further understand the biological roles of USP35 in cancer cells, USP35 expression plasmid (pcDNA3.1-USP35) was transfected into H1299 and LNCaP cells, while USP35 expression was knocked down using USP35 specific shRNAs in Hela and LNCaP cell lines. The overexpression and knock-down of USP35 were confirmed by Western blot (Figure 3A).

Next, we examined the cell growth and colony formation in the cells with overexpression and knock-down of USP35 by MTT and colony formation assay. MTT results showed that overexpression of USP35 inhibited cell proliferation significantly compared with their respective controls in both H1299 and LNCaP cells (Figure 3B). In contrast, HeLa and LNCaP cell lines with USP35 knockdown showed a significant increase of cell proliferation (Figure 3C). Moreover, colony formation analysis showed that ectopic expression of USP35 in H1299 and LNCaP cells led to significant reduced colonies numbers compared with their control cells (Figure 3D). In contrast, silenced expression of USP35 caused an increase of the numbers of colonies in HeLa and LNCaP cells (Figure 3E). These results suggest that USP35 can inhibit cancer cell growth efficiently.

### USP35 impairs the tumorigenesis in nude mice

**Figure 4:**
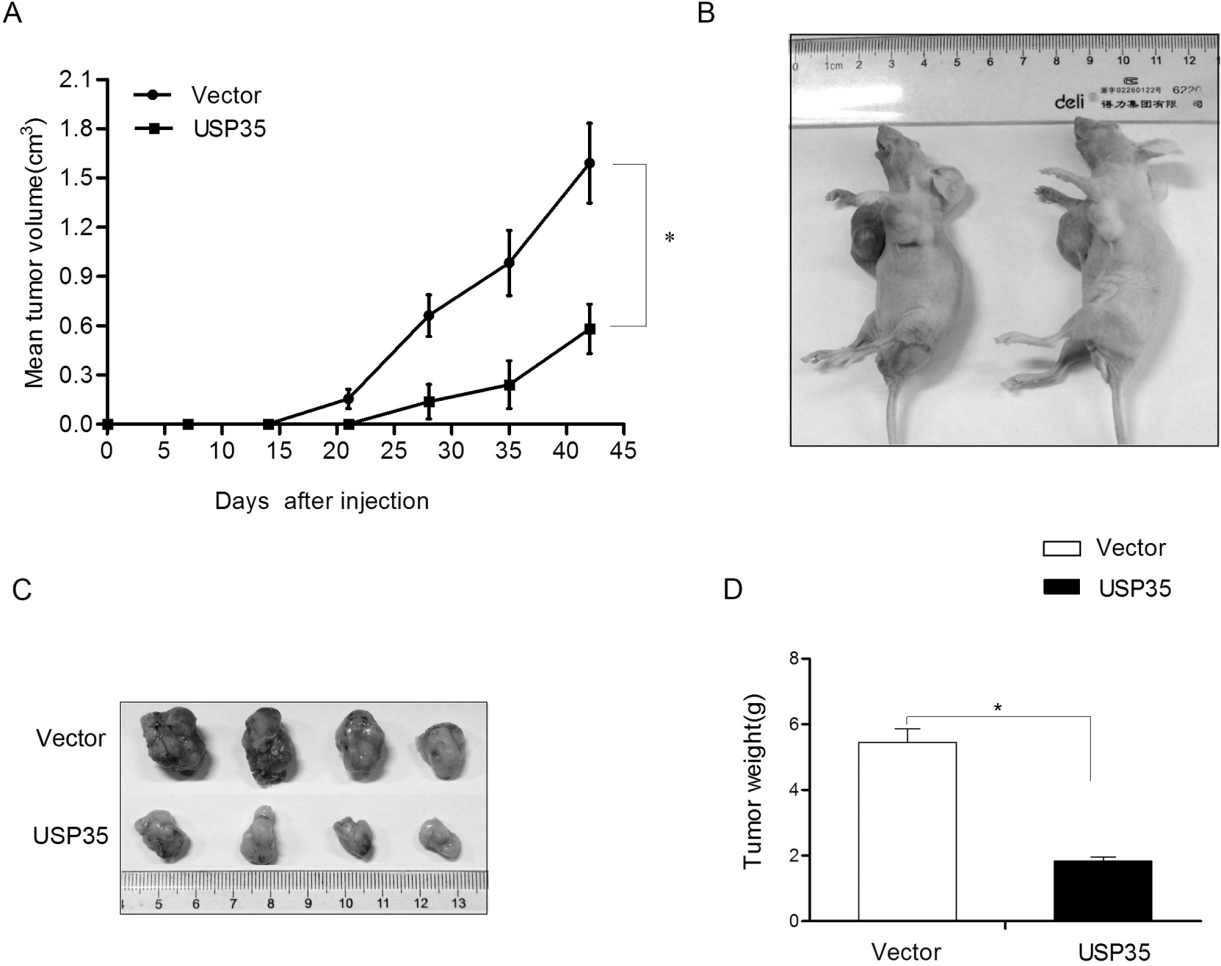
USP35 inhibits the tumor formation ability *in vivo* **A.** The volumes of the subcutaneous tumors were measured weekly with a vernier caliper after implantation. **B.** The nude mice (*n* = 6) were subcutaneously injected with H1299 cells transfected with empty vector (right) and USP35 expression plasmids (left) in the two flanks region. After 6 weeks, the nude mice were sacrificed. **C.** The images of the dissected tumors are shown. A ruler is used to indicate the size of the tumor. **D.** The tumor weight with different transfection was shown. *P < 0.05 based on Student *t-test*.

To extend our *in vitro* observations, we investigated whether USP35 could regulate tumorigenic capacity of cancer cells *in vivo*. H1299 cells with overexpression of USP35 (H1299-USP35) and its control cells were subcutaneously injected into 6 nude mice. The subcutaneous tumor size was measured every 7 days and the tumor volume was calculated. The result illustrated in Figure 4A showed that the tumors originating from the H1299-USP35 cells grew more slowly than its control cells at the implantation site. After 6 weeks, the mice were sacrificed. We found that the volume and the weight of xenografted tumor originating from the H1299-USP35 cells was much smaller and lighter than that from the control cells (Figure 4B–4D). Especially, there is no tumor formation in two mice injected with USP35-transfected cells at the end of the observation period. Taking together, these results suggested that USP35 is an inhibitor of proliferation in cancer cells both *in vitro* and *in vivo*.

### USP35 acts as a functional DUB and interacts with ABIN-2

**Figure 5:**
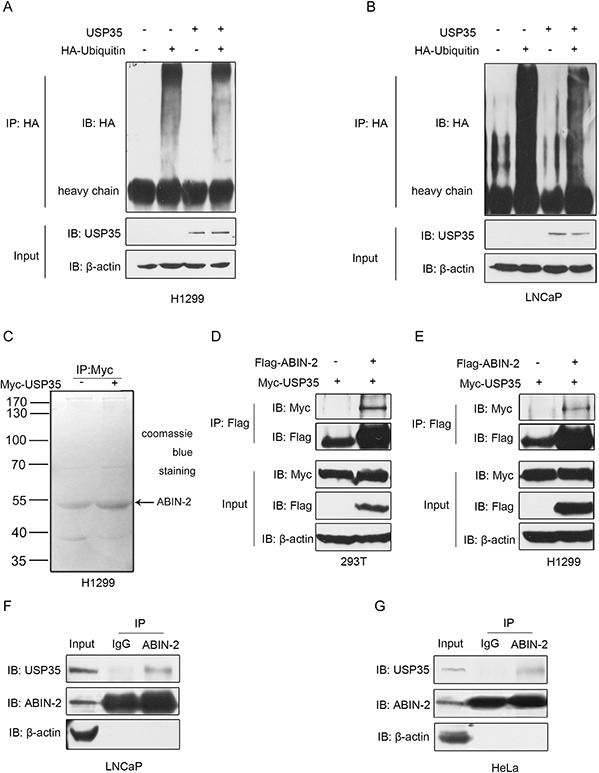
USP35 acts as a functional DUB and interacts with ABIN-2 **A. and B.** H1299 (A) and LNCaP (B) cells were transfected with HA-ubiquitin alone or together with USP35 expression plasmids. Cell lysates were immunoprecipitated with anti-HA and analyzed by immunoblotting with anti-HA. Lower panel shows the input levels of the indicated proteins. **C.** H1299 cells were transiently transfected with empty vector and plasmid expressing Myc-USP35. Cell lysates were immunoprecipitated with anti-Myc antibody and then seperated by SDS-PAGE. After staining with Coomassie blue R-250, a specific protein band was subsequently excised to mass spectrometric analysis. **D. and E.** HEK293T (D) and H1299 (E) cells were cotransfected with Flag-ABIN-2 and Myc-USP35. Cell lysates were immunoprecipitated with anti-Flag, and subjected to subsequent immunoblotting with anti-Myc or anti-Flag. Lower panel shows the input levels of the indicated proteins. **F. and G.** Whole cell lysates from LNCaP (F) and HeLa cells (G) were immunoprecipitated with anti-ABIN-2 or normal IgG, and then immunoblotted with anti-USP35 or anti-ABIN-2. Left panel shows the input levels of the indicated proteins. All results are representative of three independent experiments.

Since USP35 is a member of DUB family, USP35 has been suggested to play a critical role in regulation of protein deubiquitination. To clarify the role of USP35 in deubiquitination, LNCaP and H1299 cells were transfected with HA-tagged ubiquitin (HA-Ub) alone or in combination with pcDNA3.1-USP35 without treatment of MG132. The whole proteins were then immunoprecipitated and immunoblotted with anti-HA antibody. The results showed that overexpression of USP35 markedly reduced the ubiquitination of whole proteins (Figure 5A and 5B). To identify the specific substrates recognized by USP35, H1299 cells were transiently transfected with Myc-tagged USP35 expression plasmid and empty vector respectively. Subsequently, several affinity-purified USP35- associated factors were separated and detected by immunoprecipitation with anti-Myc antibody and mass spectrometry analysis, of which ABIN-2 was identified as a potential USP35-interacting protein (Figure 5C).

To validate the interaction between USP35 and ABIN-2, HEK293T cells were transiently transfected with Myc-tagged USP35 alone or in combination with Flag-tagged ABIN-2. Lysates were immunoprecipitated with anti-Flag antibody and then probed for USP35 by anti-Myc antibody. Western blot analysis revealed that Myc-tagged USP35 coimmunoprecipitated with Flag-tagged ABIN-2, indicating a physical interaction between the two proteins (Figure 5D). Similar results were observed in H1299 cells with exogenous expression of related proteins (Figure 5E). Further, to examine if endogenous USP35 could bind to endogenous ABIN-2, lysates of LNCaP cells were immunoprecipitated with anti-ABIN-2 antibody and probed for USP35 by anti-USP35 antibody. Western blot revealed that USP35 coprecipitated with endogenous ABIN-2 (Figure 5F). In addition, endogenous USP35 binding to ABIN-2 was also observed in HeLa cells (Figure 5G). These results indicate that ABIN-2 is a novel binding partner of USP35.

### USP35 deubiquitinates and stabilizes ABIN-2 protein

**Figure 6:**
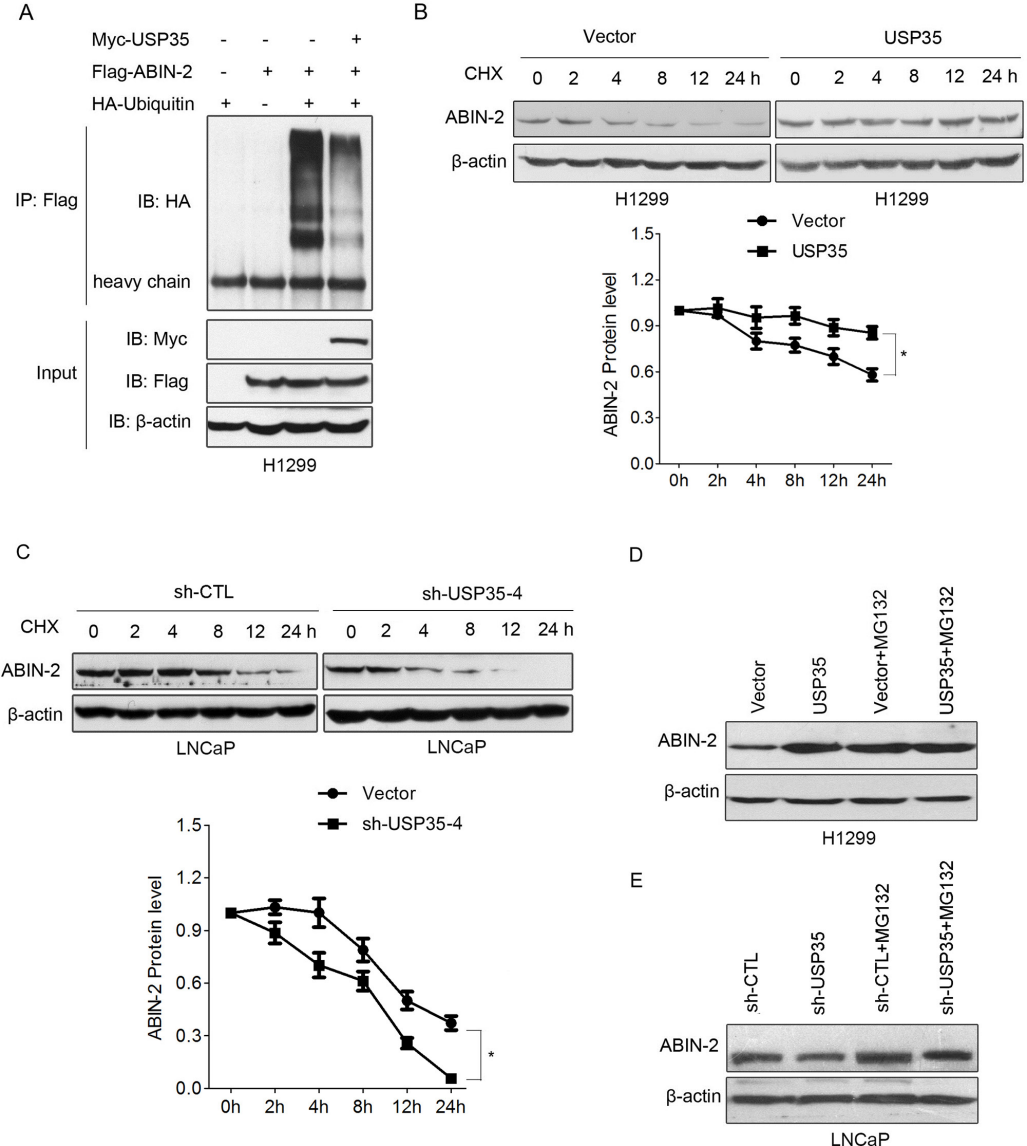
USP35 deubiquitinates and stabilizes ABIN-2 protein **A.** H1299 cells were cotransfected with Flag-ABIN-2, HA-Ubiquitin and Myc-USP35 or empty vector. H1299 cells transfected with Flag-ABIN-2 or HA-Ubiquitin alone were used as controls. Cell lysates were immunoprecipitated with anti-Flag and analyzed by immunoblotting with anti-HA. Lower panel shows the input levels of the indicated proteins. **B. and C.** H1299 cells transfected with control vector or USP35 expression plasmids (B) and LNCaP cells transfected with control shRNA or USP35 specific shRNA (C) were treated with 50 μg/ml CHX for 0, 2, 4, 8, 12 and 24 h. Western blot analysis was done to detect endogenous level of ABIN-2 using anti-ABIN-2 antibody. The graph shows quantitative analysis of the CHX chase data. **D. and E.** H1299 cells transfected with USP35 expression plasmids (D) and LNCaP cells transfected with USP35 specific shRNA were treated with or without 10 μM MG-132 for 6 h. Western blot analysis was done using anti-ABIN-2. All results are representative of three independent experiments. *P < 0.05 based on Student *t-test*.

Evidence has shown that ABIN-2 can be degraded by the ubiquitin-proteasome pathway [21]. To investigate the effect of USP35 on ABIN-2 ubiquitination, the ubiquitination of ABIN-2 was detected using an *in vivo* ubiquitination assay. HA-tagged ubiquitin and Flag-ABIN-2 were cotransfected into H1299 cells in the presence of wild-type Myc-USP35 or control vector. Immunoprecipitation with Flag-ABIN-2 followed by HA-Ub immunoblotting showed overexpression of USP35 dramatically decreased the ABIN-2 ubiquitination (Figure 6A). A similar inhibitory effect was also observed in LNCaP and HEK293T cells (Supplementary Figure S3A and S3B). These results suggest that USP35 can facilitate the deubiquitination of ABIN-2.

Next, we evaluated whether USP35 could regulate ABIN-2 protein stability. The turnover of ABIN-2 in H1299-USP35 or LNCaP-shUSP35 and their corresponding control cells was measured respectively by cycloheximide (CHX) chase assay. The results showed that overexpression of USP35 significantly extended the half-life of ABIN-2, while silenced expression of USP35 shortened the half-life of ABIN-2, indicating that USP35 promotes ABIN-2 protein stabilization (Figure 6B and 6C). Then we investigated if USP35 could protect ABIN-2 from proteasome-dependent degradation. H1299 cells with USP35 overexpression or LNCaP cells with USP35 knockdown were treated with or without proteasome inhibitor MG132. The following Western blot analysis showed that USP35 overexpression increased ABIN-2 expression while USP35 knockdown decreased its expression. Meanwhile, proteasome inhibition caused a significant increase in ABIN-2 protein level and blocked the USP35-induced different expression of ABIN-2 (Figure 6D and 6E). These data illustrated that USP35 could promote ABIN-2 deubiquitination and decrease its proteasomal degradation.

**Figure 7:**
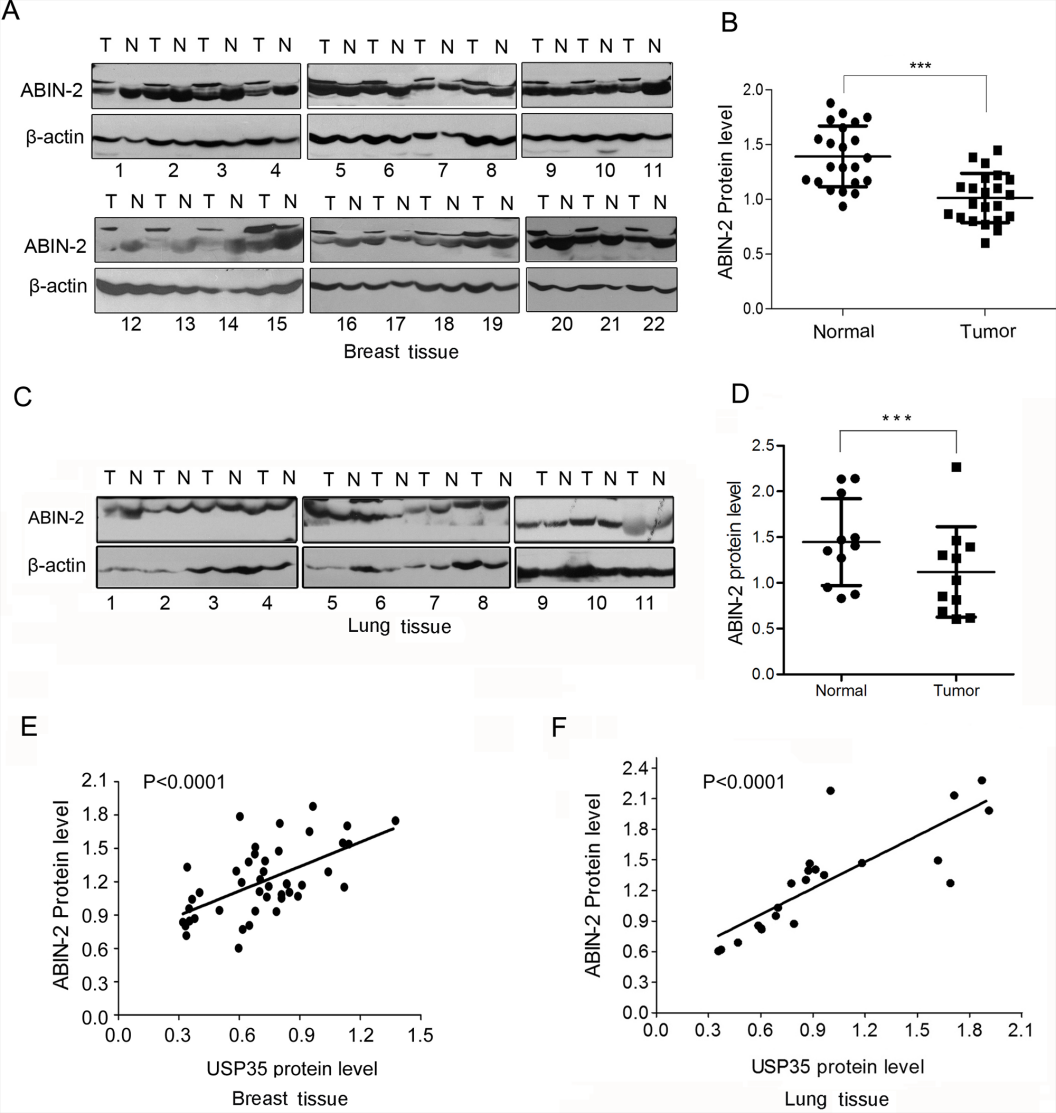
ABIN-2 expression is down-regulated in cancer tissues and positively correlated with USP35 expression **A.** The protein of ABIN-2 was detected by Western blot in breast cancer tissues and adjacent non-cancerous tissues. N and T: normal and tumor tissues. **B.** Relative ABIN-2 protein expression levels in breast cancer tissues and non-cancerous tissues (*n* = 22) were shown as scatter diagram. Data, mean ± SD. **C.** The protein of ABIN-2 was detected by Western blot in lung cancer tissues and adjacent non-cancerous tissues. N and T: normal and tumor tissues. **D.** Relative ABIN-2 protein expression levels in lung cancer tissues and non-cancerous tissues (*n* = 11) were shown as scatter diagram. Data, mean ± SD. **E.** Regression analysis of the correlations between the expression of USP35 and ABIN-2 expression in breast tissues. **F.** Regression analysis of the correlations between the expression of USP35 and ABIN-2 expression in lung tissues. ***P < 0.001 based on Student *t-test*.

Finally, we examined the expression of ABIN-2 in above-mentioned clinical samples and analyzed the correlation between USP35 and ABIN-2. ABIN-2 proteins are coordinately decreased in the most of cancer tissues (Figure 7A–7D; Supplementary Figure 4SA–4SD). The expression of ABIN-2 showed a positive correlation with the expression of USP35 (Figure 7E and 7F). Together, these data illustrated that USP35 could up-regulate the expression of ABIN-2 by promoting ABIN-2 deubiquitination and decreasing its proteasomal degradation.

### USP35 inhibits TNFα-induced NF-κB activation through stabilizing ABIN-2 protein

**Figure 8:**
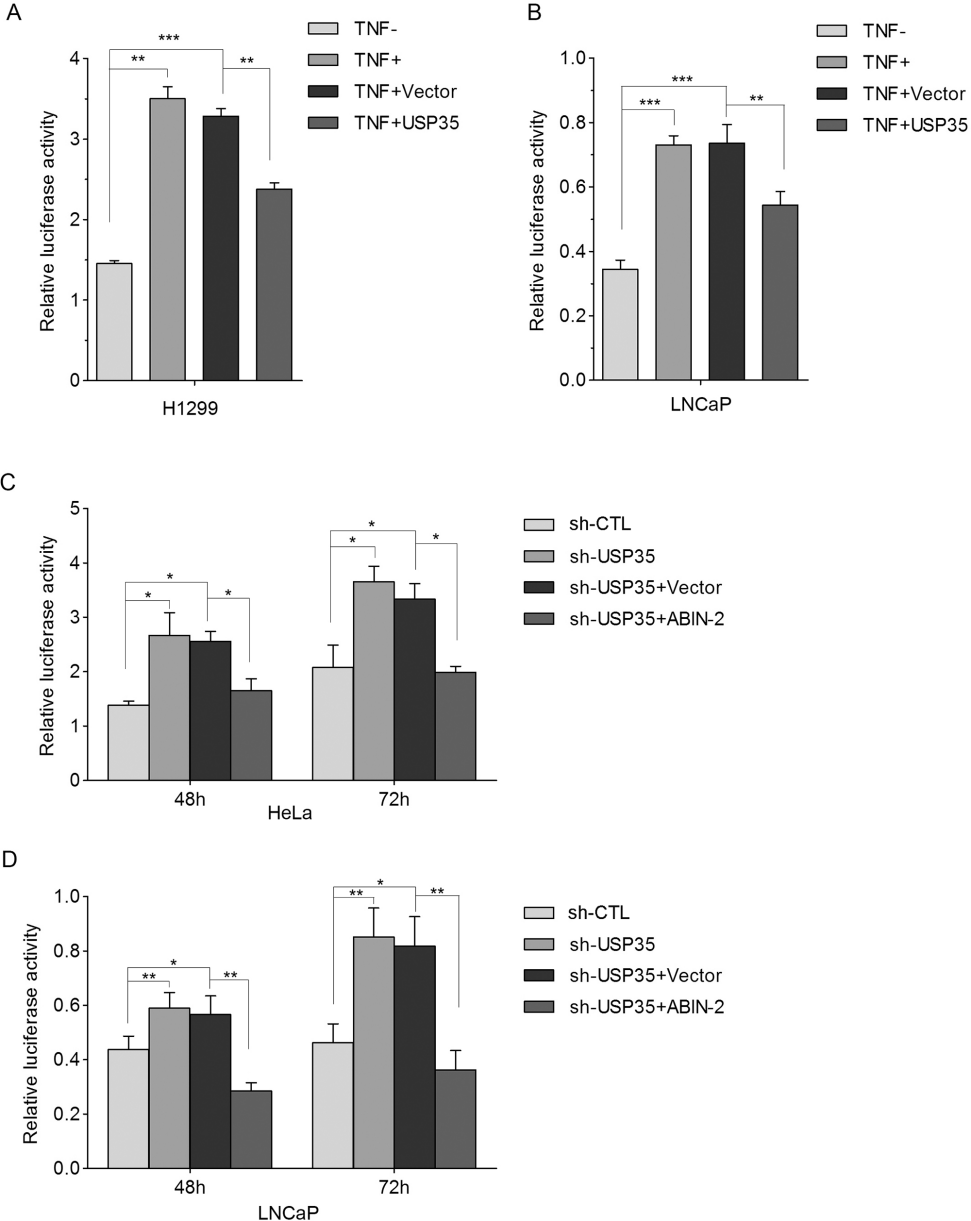
USP35 inhibits TNFα-induced NF-κB activation through stabilization of ABIN-2 **A. and B.** H1299 (A) and LNCaP (B) cells were transfected with NF-κB-dependent firefly luciferase reporter alone, or together with USP35 expression plasmids for 42 h, followed by treatment with or without TNFα (2ng/ml). 6 h later, luciferase activity was determined and was normalized to Renilla luciferase activity. **C. and D.** HeLa (C) and LNCaP (D) cells were cotransfected with NF-κB-dependent firefly luciferase reporter together with USP35 shRNA alone or with USP35 shRNA and ABIN-2 expression plasmids. Relative luciferase activity was determined to assess the influence of ABIN-2 on USP35-loss induced NF-κB activation. All results are from three independent experiments. *P < 0.05, **P < 0.01, ***P < 0.001 based on Student *t-test*.

Since it is reported that ABIN-2 could inhibit TNFα-induced NF-κB activation in HEK293T cells [22], we first verified such effect of ABIN-2 in H1299 cells (Supplementary Figure S5). Then, we investigated whether USP35 is involved in the regulation of TNFα-induced NF-κB activation using NF-κB-dependent luciferase reporter gene assay. The results showed that TNFα-induced NF-κB activation was significantly inhibited by overexpression of USP35 both in LNCaP and H1299 cells (Figure 8A and 8B). In contrast, silenced USP35 expression in LNCaP and HeLa cells extremely enhanced TNFα-induced NF-κB activation. Overexpression of ABIN-2 alleviated USP35-loss induced enhancement of NF-κB activation (Figure 8C and 8D). Taken together, these results suggest that USP35 could inhibit TNFα-induced NF-κB activation by stabilizing ABIN-2 protein.

## DISCUSSION

Accumulating data showed that MiR let-7a functions as an important tumor suppressor and plays an important role in tumorigenesis. Decreased miR let-7a expression has been linked to increased tumorigenicity and poor patient prognosis in several cancers, including lung cancer and prostate cancer [2, 23]. However, the precise mechanisms of let-7a in the pathogenesis of cancer are still incompletely understood. In the present study, to further elucidate the mechanisms underlying miR let-7a function in tumorigenesis, we globally screened the differentially expressed genes induced by miR let-7a mimics or inhibitor in LNCaP cells using cDNA microarray. Among the genes whose expression was altered by changing the level of miR let-7a, USP35 was first identified and validated with further qRT-PCR and Western blot in different cancer cell lines.

USP35 located at chromosomal band 11q14.1 and is a DUB that belongs to the ubiquitin-specific protease family. Recently, more and more studies have revealed that deubiquitination mediated by DUBs is comparable with ubiquitination involving in regulating diverse biological processes [24, 25]. DUBs were often found to be significantly dysregulated in various types of cancers and their alteration contributes to tumor development. For example, USP2a is overexpressed in prostate cancer and correlates with tumor progression and poor prognosis in oral squamous cell carcinoma [26, 27]. On the contrary, USP33 expression is downregulated in colorectal cancer samples [28] and multiple cohorts of lung cancer patients [29]. Reduced USP33 mRNA levels are correlated with increased tumor grade, lymph node metastasis and poor patient survival. Accumulating evidences highlight the importance of DUBs, however, the biological function of only a small proportion of DUBs has so far been characterized. To date, the biological roles of USP35 are mostly unknown and the molecular mechanisms underlying USP35-mediated biological activities remain largely undetermined.

In this study, we first show that USP35 has clinical significance and plays a functional role in human cancers. USP35 expression is frequently decreased in human cancer tissues when compared with adjacent normal tissues. Ectopic USP35 expression in cancer cells inhibited cell proliferation, while silencing USP35 induced proliferation *in vitro*. The growth inhibitory role of USP35 was also verified *in vivo*. Overexpression of USP35 significantly inhibited the tumorigenic potential of cancer cells in nude mice. These data suggest that USP35 plays a tumor suppressor role in tumor formation.

Consistent with our findings from the microarray analysis, USP35 expression was positively correlated with miR let-7a expression in human cancer tissues. A number of studies have proved that miRNAs including miR let-7a exerts their roles mainly through interacting with the complementary sequences in the target mRNA to induce translational repression or target degradation [30]. Several other researches have also revealed that miRNAs likewise induce translation up-regulation of target mRNAs, indicating an unanticipated versatility of microRNA function [31–34]. Although, USP35 is not a predicted target of miR let-7a by the web-based bioinformatic analysis, the fact from our experiment is that the expression of USP35 can be up-regulated by miR let-7a. The molecular pathways by which miR let-7a up-regulates USP35 need to be further investigated.

As USP35 is a member of DUBs family, we further screened and identified the putative substrates of USP35. Using coomassie blue staining and mass spectrometry analysis, USP-35-associated proteins in H1299 cells were purified. Resultantly, ABIN-2 was identified in the copurified proteins and validated with further co-immunoprecipitation assay. Subsequently, we presented a serial evidence to show that USP35 could deubiquitinate and stabilize ABIN-2 in cancer cells. In keeping with this scenario, ABIN-2 protein abundance in human tumor samples correlated well with USP35 abundance.

ABIN-2 was originally identified as intracellular NF-kB inhibitory protein that bind to the ubiquitin-editing protein A20, also known as tumor necrosis factor alpha induced protein 3 (TNFAIP3) [35]. Previous work has shown that ABIN-2 plays a pivotal role in NF-κB signal pathway [36–38]. Overexpression of ABIN-2 is critical to inhibit TNFα-induced NF-κB activation [22]. So we next determined whether USP35 was involved in regulating NF-κB pathway and finally found that USP35 down-regulated TNFa-induced NF-κB activation through promoting the function of ABIN-2. It is well known that activation of NF-κB pathway can promote cellular proliferation and oncogenic conversion [39] [40]. So we speculate that USP35 can inhibit cancer cell growth through inhibiting NF-κB pathway by stabilizing ABIN-2. However, the detailed mechanism needs to be further studied.

Collectively, the data presented here suggested that USP35, a member of DUBs family, exerts tumor-suppressive function and inhibits tumor growth which can be up-regulated by the miR let-7a in human cancers. Furthermore, USP35 can increase the activity of ABIN-2 by stabilizing its protein. Our current findings identify a regulatory network of miR let-7a-USP35-ABIN-2 pathway, which may serve as potential therapeutic targets to help us control the human cancer progression.

## MATERIALS AND METHODS

### Cell lines

Human prostate cancer cells LNCaP, PC3 and non-malignant prostate epithelial cells RWPE-1, breast cancer cells MCF7, BT474 and non-malignant breast epithelial cells MCF10A, cervical cancer cells HeLa, lung cancer cells H1299, A549 and human embryo kidney cells HEK293T were obtained from the American Type Culture Collection (ATCC, Rockville, MD). Human non-malignant cervical epithelial cells H8 and non-malignant lung epithelial cells BEAS-2B were purchased from GENNIO Biological Technology (Guangzhou, China). Cell culture was performed according to the manufacturer's protocol. The cells were maintained in humidified incubator at 37°C in an atmosphere of 95% air and 5% carbon dioxide.

### Antibodies and reagents

Rabbit polyclonal anti-USP35 (ab86791) was from Abcam (Cambridge, MA, USA). Mouse monoclonal anti-Flag (F3165) was from Sigma Aldrich (St Louis, MO, USA). Antibodies against HA (sc-7392), Myc (sc-40), β–actin (sc-1616) and ABIN-2 (sc-50526) were from Santa Cruz (Santa Cruz, CA, USA). Their respective horseradish peroxidase–conjugated secondary antibodies were purchased from Santa Cruz.

TNFα was the product of PeproTech (Rocky Hill, NJ, USA). Protein A/G-agarose was from Santa Cruz. MG132 and cycloheximide (CHX) were from Calbiochem (Billerica, MA, USA). Human miR let-7a mimics (UGAGGUAGUAGGUUGUAUAGUU), control mimics (UUCUCCGAACGUGUCACGUTT), miR let-7a inhibitors (AACUAUACAACCUACUACCUCA) and control inhibitors (CAGUACUUUUGUGUAGUACAA) were synthesized by RiBoBio (Guangzhou, China).

### Plasmids and transfection

*USP35* and *ABIN-2* full-length coding sequences were cloned into a 3 × FLAG, HA or Myc tagged CMV10 vector (Sigma) respectively, which were subsequently cloned into pcDNA3.1 vector (Clontech, Palo Alto, CA, USA). The NF-κB response element was subcloned into firefly luciferase reporter plasmid pGL4-minip vector (Promega, Madison, WI, USA). The shRNAs targeting to USP35

(sh-USP35–1:GCAGATGCTGACTGCCATTAG; sh- USP35–2:GCAACACATGCTATGTCAACA; sh-USP35–3:GCTGAGTTGGGCTCTTCTAGA; sh-USP35–4 GCGTC TGACTTCAGACATTGT) and the control shRNA (sh-CTL: GTTCTCCGAACGTGTCACGT) were synthesized and subcloned into the vector of pGPU6/GFP/Neo (GenePharma, Shanghai, China). All constructs were verified by DNA sequencing. X-tremeGENE HP DNA Transfection Reagent (Roche, Indianapolis, IN, USA) was used to transfect plasmid and siPORT NeoFX Transfection Reagent (Invitrogen) was used to transfect miRNA mimics or inhibitor into indicated cells. The transfection procedures followed the protocol of the manufacturer.

### Reverse transcription and qRT-PCR

Total RNA from the cells or tissues was extracted using TRIzol (Invitrogen, Carlsbad, CA, USA) according to manufacturer's protocol. The reverse transcription reaction was performed using the SuperScript II Reverse Transcriptase (Invitrogen). Quantitative real time polymerase chain reaction (qRT-PCR) was done using SYBR Green PCR Master Mix (Applied Biosystems, Foster City, CA, USA) in a total volume of 20 μl on a 7900 Real-Time PCR System (Applied Biosystems). The sequences of the primer pairs are: USP35 forward primer 5′ TGCCATTAGCAGGATGATTGA3′ and reverse primer 5′ AGCGAAACCTCGATCAAGATG 3′; GAPDH forward primer 5′-GCCGCATCTTCTTTTGCGTCGC-3′ and reverse primer 5′-TCCCGTTCTCAGCCTTGACGGT-3′. Calculation of target mRNA levels was based on the CT method and normalization to human GAPDH or U6 expression. The experiments were repeated three times in triplicate.

### Western blot analysis

Total protein was extracted from cells using RIPA [50 mM Tris (pH 7.4), 150 mM NaCl, 1% Triton X-100, 1% sodium deoxycholate, 0.1% SDS, 50 mM EDTA and a protease inhibitor cocktail (Roche)]. Then, 30 μg of protein were separated by SDS-PAGE (10%). After transferring to polyvinylidene fluoride (PVDF) membrane (Millipore, Billerica, MA), the membranes were incubated overnight at 4°C with antibodies against USP35 (1:5000), ABIN-2 (1:1000) and β-actin (1:2000). After incubation with peroxidase-coupled secondary antibody at 37°C for 2 h, bound proteins were visualized using ECL (Millipore) and detected using BioImaging Systems (UVP Inc., Upland, CA). The relative protein levels were calculated based on β-actin protein.

### Immunoprecipitation and Coimmunoprecipitation

To determine the interaction of the proteins, immunoprecipitation and co-immunoprecipitation were performed as following. Targeted cells were washed twice with ice-cold PBS, then whole-cell extracts were prepared by lysing cells in lysis buffer [20 mM Tris(pH7.5), 150 mM NaCl, 1% Triton X-100, 0.1 mM sodium orthovanadate, 25 mM β-glycerophosphate, 3 mM EDTA, 10 mg/ml leupeptin, and a protease inhibitor cocktails (Roche)]. About 1 mg cell lysates were incubated with indicated antibodies overnight at 4°C on a verticle roller. The lysates were incubated with 30 μl of Protein A/G agarose beads for 2 h and then the beads were washed 5 times with cold PBS. The immunoprecipitated proteins were then identified by western blot with corresponding primary antibodies.

### Luciferase reporter assay

Cells were seeded and cultured overnight in 24-well plates. Then, the cells were transfected with NF-κB-dependent firefly luciferase construct alone or together with indicated plasmids. The renilla luciferase construct was used to normalize firefly luciferase activity. At 42 h after transfection, 2 ng/ml of TNFα was added to the medium. The cells were incubated for another 6 h and then the cells were harvested and luciferase assays were performed using the Dual-Luciferase Reporter Assay System (Promega). The experiments were repeated three times in triplicate.

### Proliferation assay

The viability of cells was determined by assaying the reduction of 3-(4, 5-dimethylthiazol-2-yl)-2, 5-diphenyltetrazolium bromide (MTT). Briefly, cells were plated onto 96-well plates with 1 × 10^4^ cells per well. At indicated time, the medium was replaced by 100 μl medium and incubated with 10 μl MTT (5 mg/ml) for 4 h. The particle was dissolved with DMSO. The absorbance was determined at 570 nm.

### Colony formation analysis

The cells were seeded in 6-well plates at a density of 800 cells per well in complete medium. After 3 weeks of growth, the cells were fixed and stained with crystal violet (0.1%, w/v in 20 nM 4-morpholinepropanesulfonic acid). The number of colonies with more than 50 cells was counted. The experiments were repeated three times in triplicate.

### Nude mice xenograft model

Athymic BALB/c nude mice (4–5 weeks of age) were purchased from the HFK Bioscience Company (Beijing, China) and bred under specific pathogen-free conditions. The BALB/c nude mice (*n* = 6) were inoculated subcutaneously with H1299-vector cells (1 × 10^6^, suspended in 100 μL sterile PBS) in the right oxter as control group and with H1299-USP35 cells (1 × 10^6^, suspended in 100 μL sterile PBS) in the left oxter, respectively. The subcutaneous tumor size was measured every 7 days with a vernier caliper and the tumor volume was calculated by the formula (length) × (width^2^)/2. The mice were sacrificed after 6 weeks and the dissected tumors were collected and weighed. All experimental procedures were approved by the Institutional Animal Care and Use Committee of Shandong University.

### Patients

We obtained fresh tumor specimens and surrounding non-tumor tissues from 22 patients with primary breast cancer and 11 patients with lung cancer that underwent resection in Qilu Hospital of Shandong University between 2013 and 2014. Samples were stored at −80°C. The study was approved by the ethics committee of School.

### Statistical analysis

SPSS 17.0 software (SPSS Inc. Chicago, IL, USA) was used for statistical analysis. The expression differences of USP35, miR let-7a and ABIN-2 between tumor tissues and matched non-tumor tissues were analyzed with the Student's *t*-test. A Spearman correlation analysis was performed to assess the relationship between USP35 and miR let-7a/ABIN-2 expression in breast and lung cancer tissues using multivariate Cox regression analysis. The differences of cell proliferation and colony formation were assessed with the two-tailed unpaired Student's *t*-test. *P* < 0.05 was considered statistically significant.

## SUPPLEMENTARY FIGURES


