# Supplementary material for: USP35 activated by miR let-7a inhibits cell proliferation and NF-κB activation through stabilization of ABIN-2
Source: Oncotarget. 2015 Jun 26;6(29):27891–906. doi: 10.18632/oncotarget.4451 (PMC4695033; doi:10.18632/oncotarget.4451)
Supplement: Supplementary file 1 [file oncotarget-06-27891-s001.pdf]

SUPPLEMENTARY FIGURES

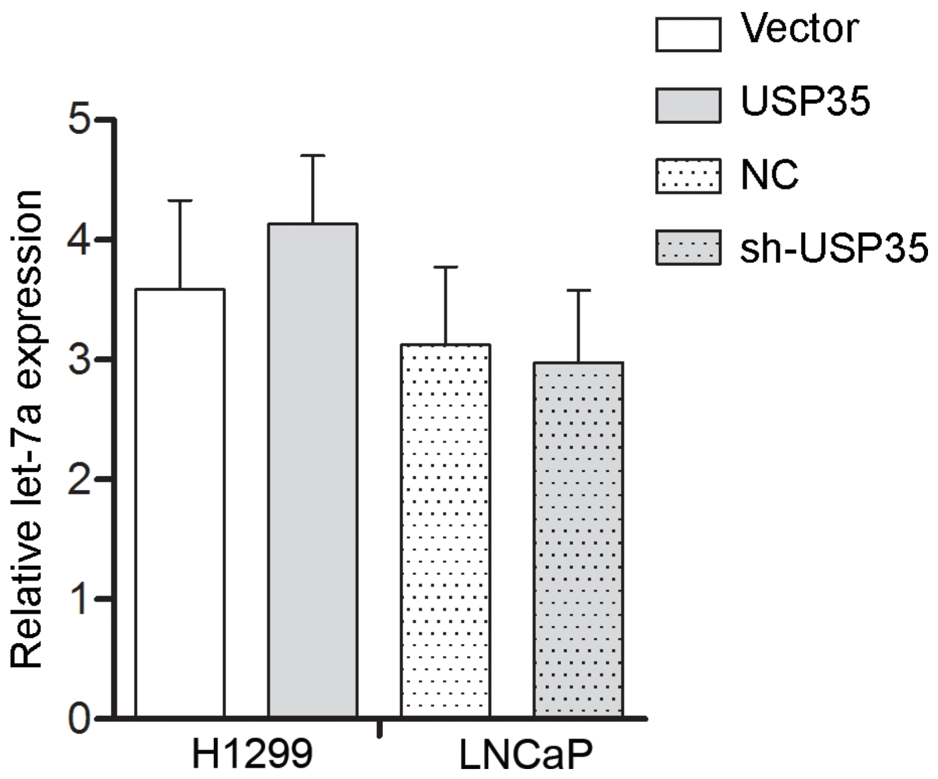

Supplementary Figure S1: USP35 has no effects on the expression of miR let-7a. MiR let-7a expression was detected by qRT-PCR in H1299 cells with USP35 overexpression and LNCaP cells with USP35 knockdown.

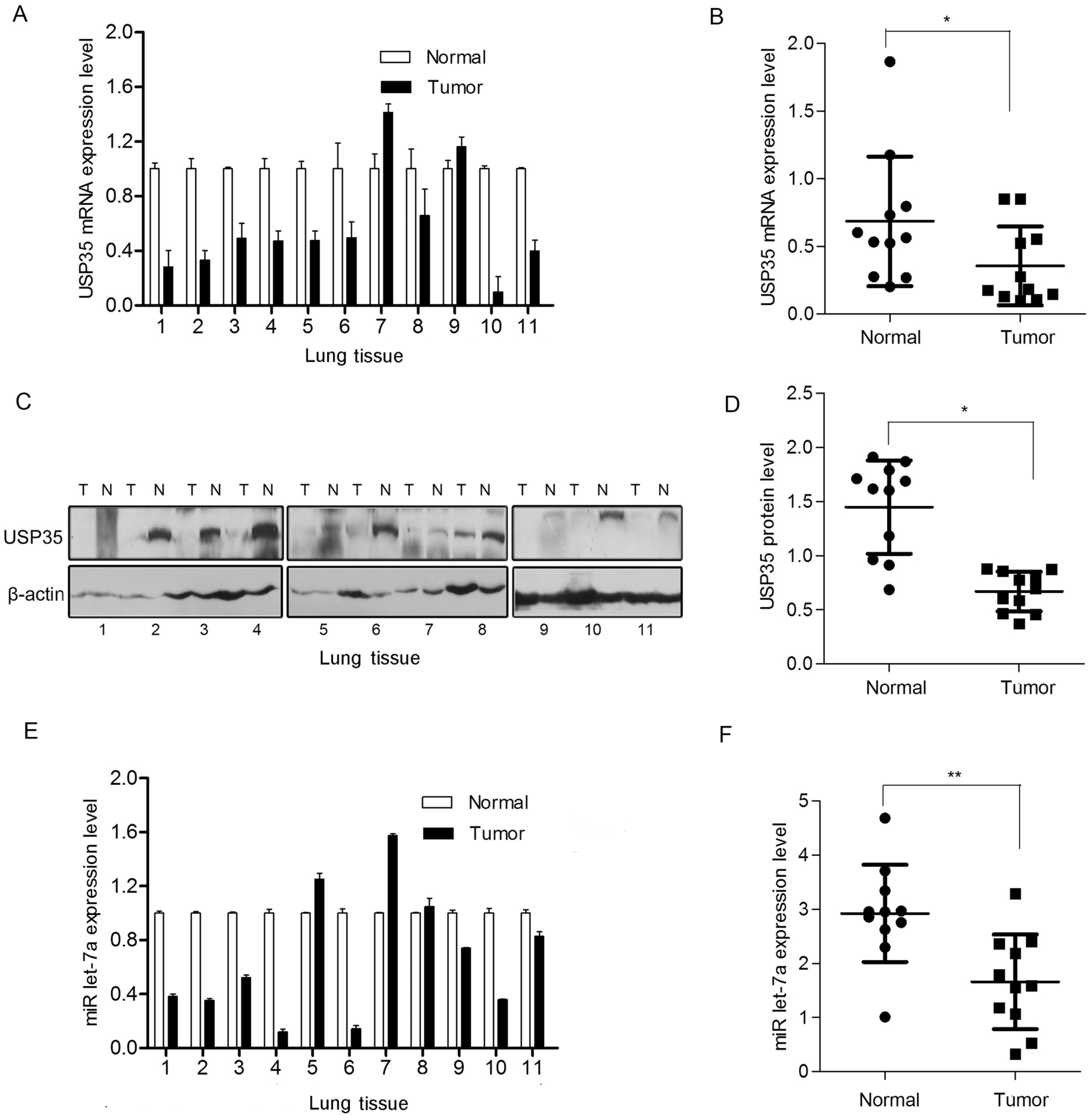

Supplementary Figure S2: USP35 and miR let-7a expression decrease in lung cancer tissues. A. The mRNA of USP35 was detected by qRT-PCR in lung cancer tissues and adjacent non-cancerous tissues. **B.** USP35 mRNA expression levels in lung cancer tissues and adjacent non-cancerous tissues (n = 11) were shown as scatter diagram. Data, mean ± SD. **C.** The protein of USP35 was detected by Western blot in lung cancer tissues and adjacent non-cancerous tissues. N and T: normal and tumor tissues. **D.** Relative USP35 protein expression levels in lung cancer tissues and non-cancerous tissues (n = 11) were shown as scatter diagram. Data, mean ± SD. **E.** The expression of miR let-7a was examined by qRT-PCR in lung cancer tissues and adjacent non-cancerous tissues. **F.** miR let-7a expression levels in lung cancer tissues and adjacent non-cancerous tissues (n = 11) were shown as scatter diagram. Data, mean ± SD. *$P < 0.05$, **$P < 0.01$ based on Student *t-test*.

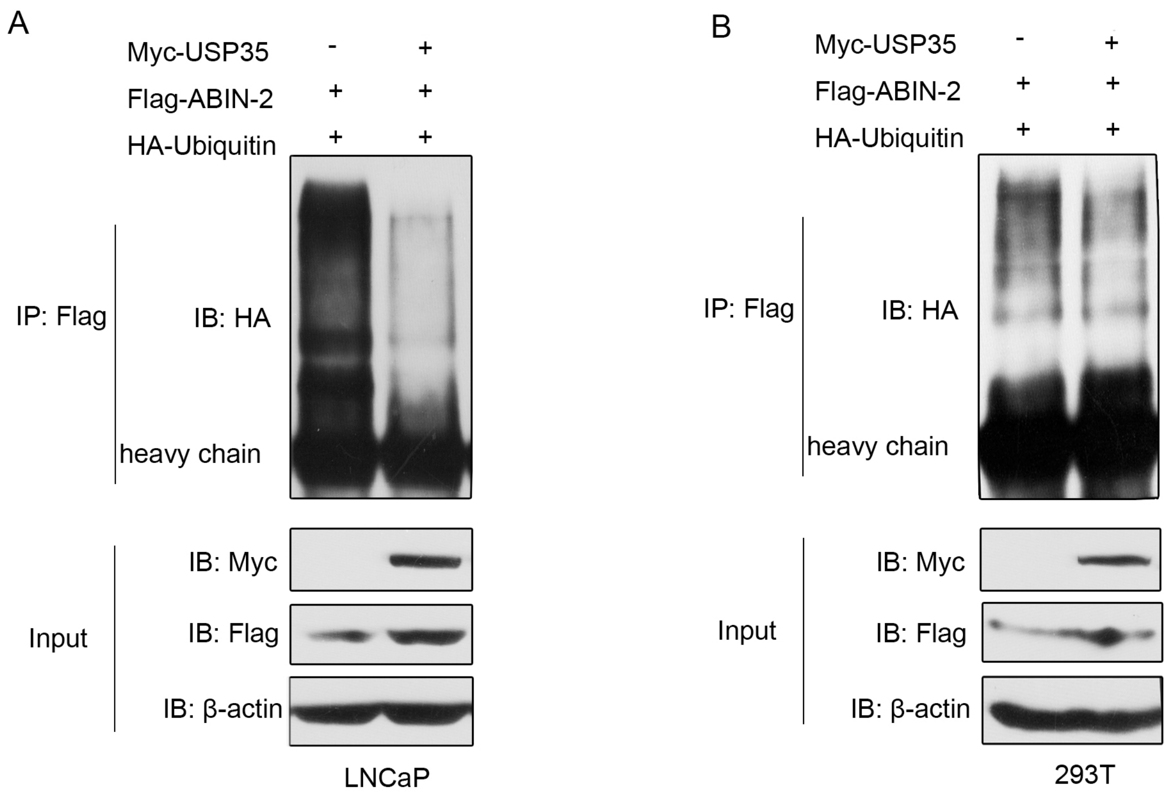

Supplementary Figure S3: USP35 deubiqutinates ABIN-2. A. LNCaP and **B.** HEK293T cells were cotransfected with Flag-ABIN-2, HA-Ubiquitin and Myc-USP35 or empty vector. Cell lysates were immunoprecipitated with anti-Flag and analyzed by immunoblotting with anti-HA. Lower panel shows the input levels of the indicated proteins. All results are representative of three independent experiments.

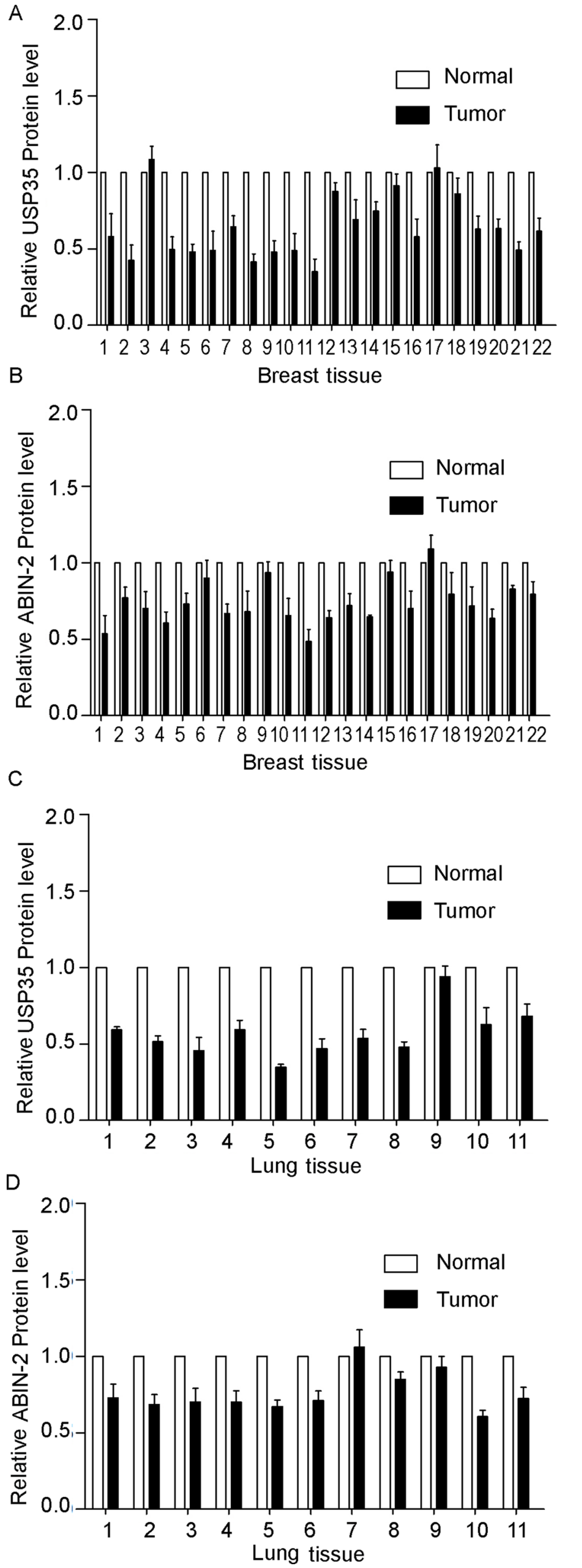

Supplementary Figure S4: The expression of USP35 and ABIN-2 were measured by Western blot in tumor and normal tissues. A. the quantification value of USP35 in breast cancer tissues and adjacent non-cancerous tissues. **B.** the quantification value of ABIN-2 in breast cancer tissues and adjacent non-cancerous tissues. **C.** the quantification value of USP35 in lung cancer tissues and adjacent non-cancerous tissues. **D.** the quantification value of ABIN-2 in lung cancer tissues and adjacent non-cancerous tissues.

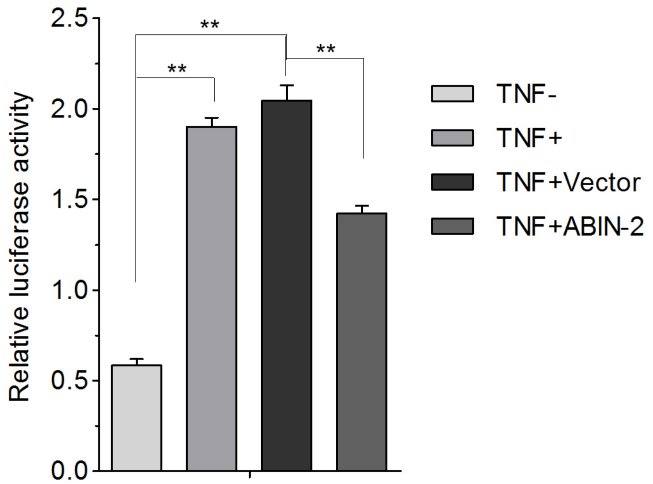

Supplementary Figure S5: ABIN-2 inhibits TNFα-induced NF-κB activation. H1299 cells were transfected with NF-κB-dependent firefly luciferase reporter alone, or together with ABIN-2 expression plasmids for 42 h, followed by treatment with or without TNFα (2ng/ml). 6 h later, luciferase activity was determined and was normalized to Renilla luciferase activity. The results are from three independent experiments. $^{**}P < 0.01$ based on Student *t-test*.
